# Pathways of N removal and N_2_O emission from a one-stage autotrophic N removal process under anaerobic conditions

**DOI:** 10.1038/srep42072

**Published:** 2017-02-16

**Authors:** Kai Li, Fang Fang, Han Wang, Chao Wang, Youpeng Chen, Jinsong Guo, Xixi Wang, Fuyang Jiang

**Affiliations:** 1 Key Laboratory of Three Gorges Reservoir Region’s Eco-Environments of MOE, Chongqing University, Chongqing 400045, China; 2 Key Laboratory of Reservoir Aquatic Environment, Chongqing Institute of Green and Intelligent Technology, Chinese Academy of Sciences, Chongqing 400714, China

## Abstract

To investigate the pathways of nitrogen (N) removal and N_2_O emission in a one-stage autotrophic N removal process during the non-aeration phase, biofilm from an intermittent aeration sequencing batch biofilm reactor (SBBR) and organic carbon-free synthetic wastewater were applied to two groups of lab-scale batch experiments in anaerobic conditions using a ^15^N isotopic tracer and specific inhibitors, respectively. Then, the microbial composition of the biofilm was analysed using high-throughput sequencing. The results of the ^15^N isotopic experiments showed that anaerobic ammonium oxidation (Anammox) was the main pathway of N transformation under anaerobic conditions and was responsible for 83–92% of N_2_ production within 24 h. Furthermore, experiments using specific inhibitors revealed that when nitrite was the main N source under anaerobic conditions, N_2_O emissions from heterotrophic denitrification (HD) and ammonia-oxidizing bacteria (AOB) denitrification were 64% and 36%, respectively. Finally, analysing the microbial composition demonstrated that Proteobacteria, Planctomycetes, and Nitrospirae were the dominant microbes, corresponding to 21%, 13%, and 7% of the microbial community, respectively, and were probably responsible for HD, Anammox, and AOB denitrification, respectively.

Nitrous oxide (N_2_O), a powerful greenhouse and ozone-depleting gas, has a lifetime of approximately 118 to 131 years and is 300–fold more potent than carbon dioxide (CO2)^1^,^2^. N_2_O contributes 6 to 8% of the anthropogenic greenhouse effect worldwide^3^. Moreover, the atmospheric concentration of N_2_O has increased at an annual rate of 0.2 to 0.3% over the past decade^4^. N_2_O can be produced in biological wastewater treatments, especially treatments involving biological nitrogen (N) removal^5^,^6^. Recently, wastewater treatment plants (WWTPs) were found to exhibit gradually rising N_2_O emissions due to increases in population density and industrial activity^7^. Therefore, studying the N_2_O emissions of biological N removal systems is beneficial for controlling the greenhouse effect and protecting the ozone layer.

The one-stage autotrophic N removal process is especially well suited for treating wastewater containing high ammonia but low organics, such as landfill leachate, livestock wastewater and agricultural effluent^8^, because it has several advantages: a low demand for aeration, no consumption of organic carbon and low sludge production^9^,^10^. In a spatial model of biofilm from a one-stage completely autotrophic N removal process, ammonia-oxidizing bacteria (AOB) and anaerobic ammonium-oxidizing bacteria (AnAOB) grew in different regions according to the concentration of dissolved oxygen (DO)^11^. In this case, ammonia was initially oxidized to nitrite by AOB located in an area of higher DO, i.e., the surface of the biofilm. Then, the nitrite and remaining ammonia are converted to N_2_ by AnAOB in anaerobic zones^8^. Kartal *et al*.^12^ presented Eq. 1 to describe the Anammox process.


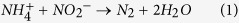


Ammonium is the main N source during one-stage autotrophic N removal. Meanwhile, nitrite is produced by the oxidation of ammonia, and N_2_ forms through the pairing of one N atom from ammonium and another N atom from nitrite^13^. Although the Anammox process is not fully understood, it is generally thought to produce no N_2_O gas^14^,^15^. Thus, improving the Anammox activity would be beneficial for reducing N_2_O emissions. However, the Anammox activity and its contribution to the removal of total N (TN) have not been measured in one-stage autotrophic N removal, making reducing the N_2_O generated in this process difficult.

In addition, heterotrophic denitrifying bacteria were also found in the systems used to treat wastewater containing high levels of ammonia-N without organics^16^, which suggests that heterotrophic denitrification (HD) is likely an additional pathway for N removal in the one-stage autotrophic N removal process. Traditionally, AOB denitrification and HD have been considered the two main pathways responsible for N_2_O emissions from biological N removal processes when DO is limited^17^,^18^. The presence of AOB and HD bacteria in the system indicates that the one-stage autotrophic N removal process might be a potential source of N_2_O emissions. In HD, N_2_O is believed to be an intermediate produced during denitrification that can be converted into N_2_ by nitrous oxide reductase (N_2_OR)^19^. In contrast, AOB denitrification is thought to contribute the same level of N_2_O emissions as HD, or perhaps more, in terrestrial and marine ecosystems because of the lack of genes encoding traditional N_2_OR^20^,^21^. Typically, AOB denitrification can be influenced by the concentration of DO or elevated nitrite^22^,^23^, where as HD is closely related to nitrite accumulation, oxygen inhibition and the presence of biodegradable organic compounds^24^,^25^,^26^.

However, the contributions of AOB denitrification and HD to N_2_O emissions when the one-stage autotrophic N removal processis used to treat high-ammonia-N, organic-free wastewater remains unclear, especially under anaerobic conditions, such as non-aeration during the application of intermittent aeration or the inner space of the micro-biofilm environment when limited oxygen is supplied to the bulk liquid. Clearly, the emission of N_2_O under anaerobic conditions is an important contribution of the total N_2_O emissions of this system. Therefore, better understanding these mechanisms is essential for formulating operating strategies to minimize N_2_O.

This study was conducted to investigate the pathways of N removal and N_2_O emission from a one-stage autotrophic Nitrogen removal process under anaerobic conditions. Biofilm from a sequencing batch biofilm reactor (SBBR) was used for two groups of batch experiments, and the microbial composition was analysed. First, an ^15^N isotope tracer technique was applied to investigate the contributions of Anammox and denitrification to TN removal via a one-stage autotrophic N removal process (batch test 1). Then, the N_2_O emissions corresponding to AOB denitrification and HD were quantified using specific inhibitors in this system (batch test 2). Finally, the microbial diversity and functional microorganisms associated with N_2_O emissions were analysed via high-throughput sequencing technology.

## Results and Discussion

### Performance of N transformation in the SBBR

**Figure 1:**
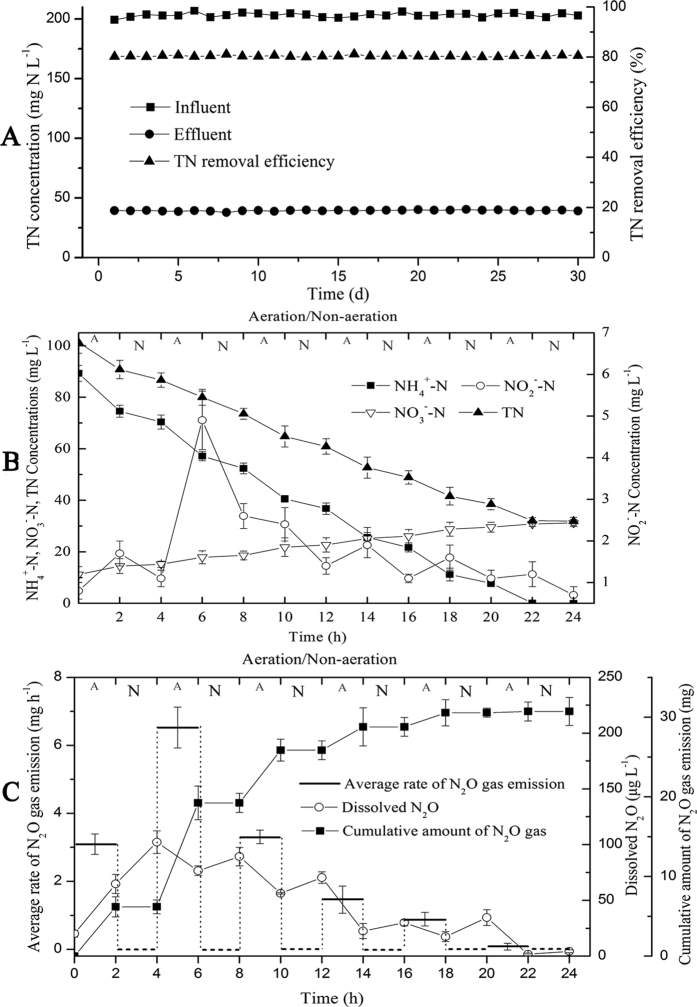
The N removal performance in the SBBR. (**A**) The TN removal efficiency during a recent month; (**B**) The concentrations of NH_4_^+^-N, NO_2_^−^-N, NO_2_^−^-N and TN in a cycle; and (**C**) the N_2_O emission in a cycle.

The SBBR operated for more than one year with a stable effluent nutrient level and TN removal efficiency exceeding 80%. Figure 1 presents the N transformation performance of the SBBR in the final month of operation. The effluent TN remained in the range of 37.9–40.4 mg N L^−1^, and the TN removal efficiency was 80.6 ± 0.6% (Fig. 1(A)). The N compounds involved in the cycle are also shown in Fig. 1(B). The NH_4_^+^-N concentration gradually decreased from 89.3 mg N L^−1^ to 0 mg N L^−1^ as NO_3_^−^-N production increased from 11.2 mg N L^−1^ to 31.2 mg N L^−1^, whereas the NO_2_^−^-N concentration did not exceed 5 mg N L^−1^ during this whole phase. In particular, NH_4_^+^-N exhibited a higher disappearance rate during aeration phases than during non-aeration followed by the increase of NO_2_^−^-N. This behaviour suggests that nitrosation occurred during the aeration phase, whereas during the non-aeration phase, NH_4_^+^-N and NO_2_^−^-N simultaneously disappeared via the Anammox process. These results indicate that nitrosation-Anammox is the main pathway for N removal in this system. However, during 22 to 24 h of NO_2_^−^-N degradation, the NH_4_^+^-N phase was completely removed, suggesting that denitrification occurred.

The N_2_O emissions corresponding to a single cycle of the SBBR are shown in Fig. 1(C). According to Eq. 6, the N_2_O-N emission factor throughout the process (EF_(total)_) was 3.3% in the SBBR, which is similar to the result reported by Liu *et al*.^27^, and 2.7% of the TN input was converted to N_2_O-N in the simultaneous nitrification-denitrification (SND) process with intermittent aeration (aeration DO:1.5–2.0 mg/L). Jia *et al*.^28^, who used a lower DO (0.35–0.80 mg/L) during the aerobic phase, found that EF_(total)_ was 7.7%. These results indicated that at the one-stage, completely autotrophic N removal and SND processes likely had similar sources of N_2_O emission, mainly during phases of low DO. However, the rates of N_2_O emission during the aeration intervals were much higher than those during the non-aerated intervals, probably because the later are associated with lower gas/liquid transfer coefficients. As a result, N_2_O emission occurs in both production processes, and stripping from the liquid arises during aerated intervals. Furthermore, the dissolved N_2_O increased during the non-aeration phase, suggesting that this phase is an important stage in N_2_O generation and may generate more N_2_O than the aeration phase. Specifically, the maximum rate of N_2_O emission was observed between 4 and 6 h, when the increase in nitrite was maximized. This finding indicates that N_2_O emission was affected by nitrite accumulation.

### Pathways of N removal

**Figure 2:**
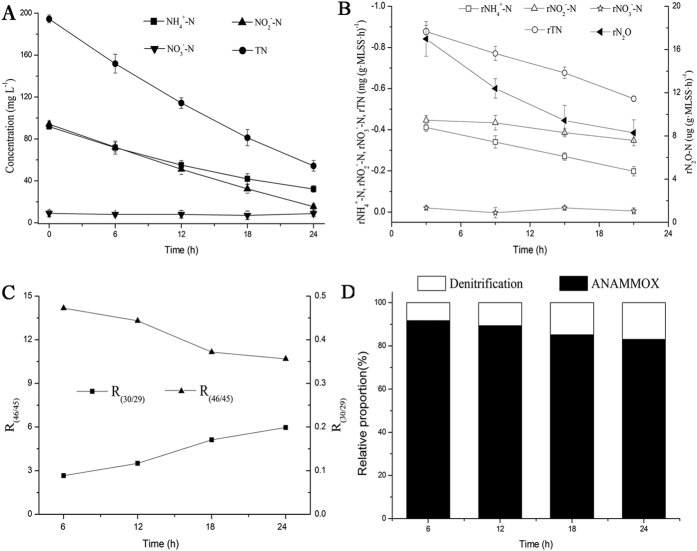
Nitrogen transformation and isotopic analysis of the batch experiments using the ^15^N-NO_2_^−^ isotopic tracer. (**A**) The concentrations of nitrogen compounds; (**B**) the rates of nitrogen transformation (the positive axis represents the apparent rate, and the negative axis represents the disappearance rate); (**C**) R_30/29_ (Square) and R_46/45_ (Triangle); and (**D**) Relative contributions of denitrification and Anammox to N_2_ production.

Table 1 shows the substrate addition strategies and N removal performances of batch test 1. The rates of TN removal in group A and group B were −0.08 mg (L h)^−1^ and 0.07 mg (L h)^−1^, respectively, and were far below that of group C (5.82 mg (L h)^−1^), reflecting both the anaerobic conditions of the experiments and the negligible effect of endogenous metabolism on TN removal. The concentrations of nitrogenous compounds and rates of N transformation (i.e., the appearance or disappearance rates of TN, NH_4_^+^-N, NO_2_^−^-N, NO_3_^−^-N, and N_2_O-N) were measured in group C. The results (Fig. 2(B)) demonstrated that the rate of disappearance of NH_4_^+^-N(rNH_4_^+^-N) decreased gradually from 0.4 to 0.2 mg (g·MLSS·h)^−1^. Meanwhile, the rate of disappearance of NO_2_^−^-N (rNO_2_^−^-N) decreased gradually from 0.5 to 0.4 mg (g·MLSS·h)^−1^. rNH_4_^+^-N and rNO_2_^−^-N displayed similar, gradually reducing trends, but rNO_2_^−^-N was always higher than rNH_4_^+^-N. The average rNH_4_^+^-N and rNO_2_^−^-N were 0.3 and 0.4 mg (g·MLSS·h)^−1^, respectively, and the related ratio of rNO_2_^−^-N to rNH_4_^+^-N was 1.34, which is similar to the Anammox stoichiometry (1.32) for this ratio according to Strous *et al*.^29^ and van der Heijden *et al*.^30^. This finding indicates that Anammox plays the main role in the N removal process. During the test, the ratio of rNH_4_^+^-N to rNO_2_^−^-N decreased gradually from 91% to 60%, indicating a gradual increase in the relative contribution of denitrification to N removal.

The value of *R*_30/29_ (Fig. 2) was determined by IRMS, and the relative contributions of Anammox and denitrification were calculated (Fig. 2(B)) via Eqs 2 and 3. The results showed that R_30/29_ gradually increased from 0.09 to 0.19; thus, 83–91% of all N_2_ was produced by Anammox, and 9–17% was generated via denitrification. These results suggested that Anammox plays the primary role in N removal, consistent with the conclusion drawn above. In addition, the relative contribution of denitrification was found to gradually increase during the operation. Previous studies have shown that autotrophs supply heterotrophs with soluble microbial products (SMPs) for use as electron donors and carbon sources^31^,^32^; subsequently, in turn, autotrophs receive inorganic carbon from heterotrophs metabolizing SMPs^33^. Therefore, the increased denitrification was probably attributable to the synthesis of SMPs, which can act as a potential electron donor for denitrification, by AOB.

### N_2_O emission under anaerobic conditions

The N_2_O-N emission from batch test 1 gradually decreased from 17.0 to 8.2 μg (g·MLSS·h)^−1^ (Fig. 2(B)), and the EF_(total)_ was 1.6%, as calculated using Eq. 6. The EF_(total)_ of batch test 1 was significantly lower than that of the SBBR (3.3%) because of the absence of nitrification, which is another source of N_2_O emission under aerobic conditions^31^,^32^. Furthermore, DO exerts an important influence on N_2_O emission from denitrification via HD bacteria and AOB^33^,^34^, and the DO concentration of batch test 1 differed substantially from that of the SBBR, which may also affect N_2_O emission. The isotopic composition of N_2_O from batch test 1was determined by IRMS (Fig. 2(C)). The results showed that *R*_46/45_ was much larger than *R*_30/29_, indicating that the pathways of N_2_O emission are quite different from those of N_2_ production. Additionally, the values of *R*_46/45_ gradually declined from 14.2 to 10.7, whereas according to Eq. 6, the ratio of *D*_*30*_ to *D*_*29*_ was equal to 26. This finding suggested that denitrification is not the only pathway to generate N_2_O. However, Anammox does not generate N_2_O. Thus, a pathway for N_2_O emission other than denitrification may exist and could potentially be an intermediate step in the denitrification process.

### N_2_O emission from AOB denitrification and HD

**Figure 3:**
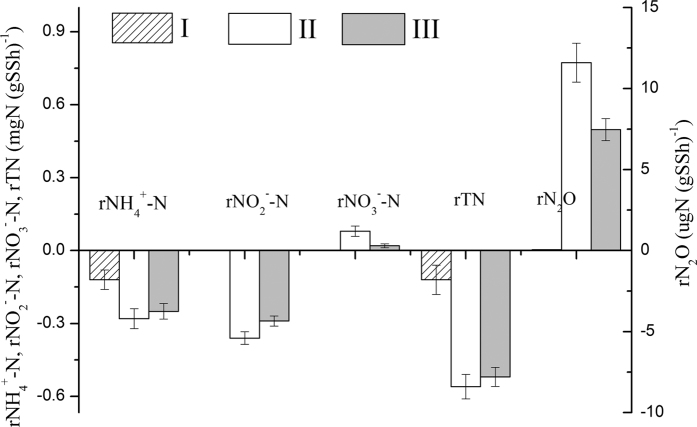
The rates of nitrogen transformation in batch experiments with inhibitors. (**I**) With no addition of nitrite or inhibitor; (**II**) with the addition of nitrite; and (**III**) with the addition of nitrite and inhibitors.

To estimate the pathways of N_2_O emission during the process of denitrification, an approach using specific inhibitors was applied to determine the proportions of the total N_2_O emission corresponding to AOB denitrification and HD. No significant N_2_O emission was observed in group I without the addition of NO_2_^−^-N and inhibitors (Fig. 3). As NO_2_^−^-N was added to system (group II), AOB denitrification and HD occurred simultaneously, and the average N_2_O-N release rate was 11.6 μg (g·MLSS·h)^−1^. Meanwhile, with the addition of inhibitors (group III), AOB denitrification was inhibited, and the average release rate of N_2_O-N was 7.5 μg (g·MLSS·h)^−1^. Thus, the release rate reduction of 4.1 μg (g·MLSS·h)^−1^ reflects the activity of AOB denitrification. Calculations based on the N_2_O emissions results showed that 36% and 64% of N_2_O emissions were from AOB denitrification and HD, respectively, during the denitrification process, implying that HD is the main pathway of N_2_O emission under anaerobic conditions.

### Microbial distributions

**Figure 4:**
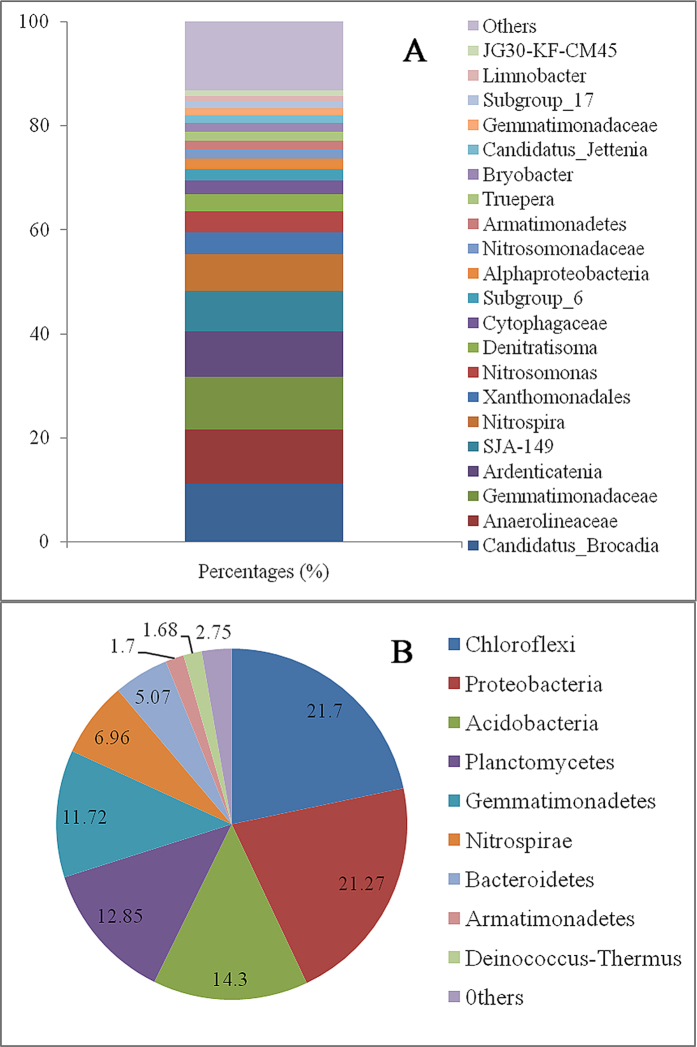
Microbial composition of the biofilm. (**A**) Sequence assignment results at the genus level; and (**B**) sequence assignment results at the phylum level. All effective sequences in the biofilm sample were assigned, and only those with high relative abundances (>0.5%) are shown in this figure.

Figure 4 presents the microbial composition of the biofilm based on the 16S rDNA amplicon pyrosequencing. These results suggest that the dominant microorganisms in the biofilm were *Candidatus brocadia*, Anaerolineaceae, Gemmatimonadaceae, *Ardenticatenia*, Nitrospira, Xanthomonadales, *Nitrosomonas* and *Denitratisoma*, with relative abundances of 11.2%, 10.4%, 10.1%, 8.7%, 7.0%, 4.2%, 4.1%, and 3.3%, respectively (Fig. 4(A)). *C. brocadia, Nitrosomonas* and *Denitratisoma* have been reported to be Anammox, AOB denitrification and HD bacteria, respectively^35^. In addition, Nitrospira has been shown to be distributed in the outer layers of biofilms and to possess the ability to convert nitrite into nitrate^36^, whereas Xanthomonadales was classified as a member of Gamma proteobacteria, which are regarded as a type of HD bacteria. However, the roles of some species in N removal remain unknown. Thus, each phylum was classified based on 16S rDNA to investigate the biological bases for N removal and N_2_O emissions (Fig. 4(B)). Chloroflexi, Proteobacteria, Acidobacteria, Planctomycetes, Gemmatimonadetes, Nitrospirae and Bacteroidetes were the main phyla. Most of the Anammox bacteria, HD bacteria and AOB for wastewater treatment could be classified as Proteobacteria, Planctomycetes and Nitrospirae, respectively^37^,^38^,^39^, which corresponded to 21%, 13%, and 7% of the total bacteria in the biofilm of this system. Thus, these bacteria might be the main sources of N_2_O emissions under anaerobic conditions.

## Conclusions

The relative contributions of denitrification and Anammoxto N_2_ production were calculated to investigate the N removal pathways in a one-stage autotrophic N removal system under anaerobic conditions. Anammox played the most important role in N removal, and denitrification emitted the most N_2_O, despite contributing little to N removal. Furthermore, HD created more N_2_O emissions than AOB denitrification under anaerobic conditions, although AOB denitrification was expected to be the more worrisome source of these emissions. Therefore, improving Anammox and decreasing denitrification contributed to reducing the N_2_O emissions of the system.

## Materials and Methods

### SBBR operation and synthetic wastewater

The SBBR consisted of a rigid Plexiglas^®^ cylinder with an effective volume of 30 L, including approximately 9 L (30%, V/V) of flexible medium for biofilm growth. The bioreactor was operated at 30 ± 2 °C with intermittent aeration (aeration:non-aeration = 2 h:2 h) and a cycle time of 24 h (i.e., 4 min of feeding, 23 h of reaction, 30 min of settling and 26 min of decanting). The DO concentration in the aeration phase was controlled at 1.5 to 2.0 mg L^−1^. In each cycle, approximately 10.5 L of wastewater was fed into the bioreactor, and the same amount of supernatant was with drawn after settling, resulting in a hydraulic retention time (HRT) of 48 h. The synthetic wastewater fed into the parent SBBR contained 1.13-g L^−1^NH_4_HCO_3_ (200-mg L^−1^NH_4_^+^-N), 583.61-mg L^−1^NaHCO_3_ and 20-mg L^−1^KH_2_PO_4_. NH_4_HCO_3_ and KH_2_PO_4_ were added as N and phosphorus sources, and NaHCO_3_ was used to regulate the pH between 7.8 and 8.2. In addition, an appropriate amount of trace elementswere added to support microorganism growth, as described by Jia *et al*.^40^.

### Isotopic tracer experiment

To distinguish the contributions of Anammox and denitrification to N removal in the one-stage autotrophic N removal process, a ^15^N-NaNO_2_ isotopic tracer was added to a sealed bottle with an active volume of 100 ml that contained 10 g wet weight of biofilm from the SBBR and 90 ml of synthetic wastewater (Table 1). The biofilm had been previously incubated for 5 h to remove nitrate from the biofilm. Next, helium gas was introduced to eliminate DO from the sealed Erlenmeyer flask containing the biofilm and pure water, and the temperature was controlled at 30 ± 2 °C. Then, the pure water was replaced with synthetic wastewater that was continuously sparged with helium gas; all other conditions remained constant. The wastewater contained 100-mgN L^−1^ NH_4_HCO_3_ and 100-mgN L^−115/14^N-NaNO_2_. ^15/14^N-NO_2_^−^ was provided by Sangon Biotech (Shanghai, China), and the ^15^N atomic percentage (100% × ^15^N/(^15^N + ^14^N), F) was 99%. The pH was controlled between 7.8 and 8.2 by the addition of NaHCO_3_. To evaluate the background N_2_O emissions from the biofilm and check the anaerobic conditions, two control groups were performed: (A) pure water and (B) synthetic wastewater with NH_4_HCO_3_ only. The off-gas was collected every 6 h for 24 h to simultaneously analyse the isotopic compositions of N_2_ and N_2_O, and 2-ml liquid samples were collected to determine the concentrations of NH_4_^+^-N, NO_2_^−^-N and NO_3_^−^-N. Finally, 100 μl of 50% ZnCl_2_ was added to the liquid samples to inhibit microbial activity.

The isotope composition of N_2_ was analysed to quantify the contributions of Anammox and denitrification to N_2_ production. In incubations with ^15^NO_2_^−^ and NH_4_^+^, N_2_ production via Anammox consisted of one N atom from NO_2_^−^ and another from NH_4_^+^, leading to the production of ^29^N_2_, whereas the denitrification of two N atoms from NO_2_^−^ was assumed to produce ^30^N_2_. However, because the F of ^15^NO_2_^−^ was not 100%, ^28^N_2_ and ^29^N_2_ were produced via Anammox, and ^28^N_2_, ^29^N_2_, and ^30^N_2_ were generated via denitrification. Therefore, the N_2_ production mass of Anammox and denitrification could be respectively calculated according to Thamdrup and Dalsgaard^41^, The calculations were described as Eqs 2 and 3:


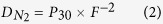



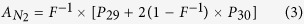


where 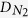 and 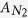 represented the mass of N_2_ produced by denitrification and Anammox, respectively; *P*_*29*_ and *P*_*30*_ represent the production amount of ^29^N_2_ and ^30^N_2_, respectively, and F represents the fraction of ^15^N in the NO_2_^−^ pool. In this system, Anammox and denitrification were the only two pathways of N removal, the relative contributions of denitrification (*Cd*) and Anammox (*Ca*) to N_2_ production can be described as the ratio of 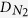 to 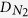 plus 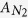 and that of 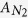 to 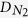 plus 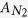, respectively. Therefore, *Cd* and *Ca* can be described by Eqs. 4 and 5, respectively:


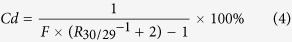



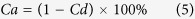


In which *R*_30/29_ represents the ratio of ^30^*N*_*2*_ production to ^29^*N*_*2*_ production

The isotopic composition of N_2_O was also investigated. N_2_O was generated as an intermediatein both nitrification and denitrification during the process of biological N removal^42^. Therefore, denitrification should be the only pathway of N_2_O emission under anaerobic conditions, and N_2_O should possess an isotopic composition similar to that of the N_2_ produced by denitrification; that is, the ratio of ^46^N_2_O to ^45^N_2_O (R_46/45_) should be equal to the ratio of ^30^N_2_ to ^29^N_2_ of denitrification. The ratio of ^30^N_2_ to ^29^N_2_ corresponding to denitrification can be expressed using Eq. 6 according to Thamdrup and Dalsgaard^41^


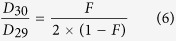


where *D*_*30*_ and *D*_*29*_ represent the production of ^30^N_2_ and ^29^N_2_ via denitrification, respectively. Thus, if R_46/45_ was not equal to the ratio of *D*_*30*_ to *D*_*29*_, denitrification was not the only pathway for N_2_O emission.

### Experiments involving specific inhibitors

The use of inhibitors can facilitate investigating the magnitudes of the various processes at the source of N_2_O production under anaerobic conditions. Allylthiourea (ATU) was used as the inhibitor of the nitrification of ammonia to nitrite, whereas NaClO_3_was used to inhibit the conversion of nitrite to nitrate catalysed by nitrite oxido-reductase^28^. The co-use of ATU and NaClO_3_ can effectively inhibit the production of N_2_O via AOB denitrification^37^, whereas N_2_O emissions by heterotrophic bacteria are not significantly affected by the presence of ATU and NaClO_3_^37^. Therefore, the emission of N_2_O produced by HD alone and by both AOB denitrification and HD can be quantified by batch experiments with or without the inhibitors.

**Figure 5:**
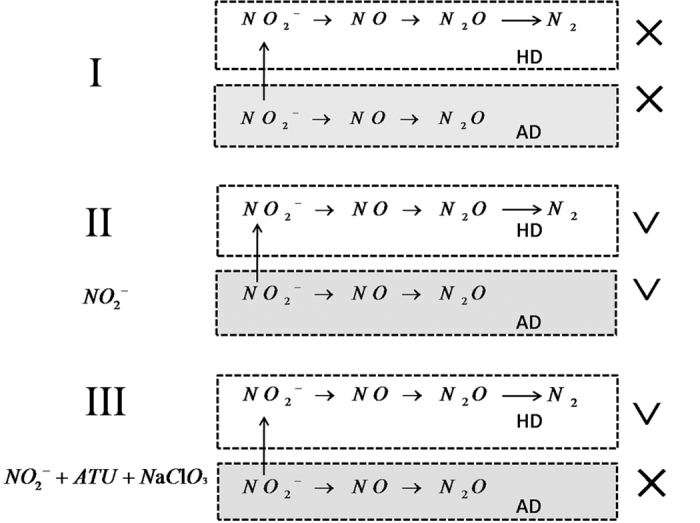
Diagrams of expected nitrogen transformation under anaerobic conditions. (**I**) No NO_2_^−^-N or inhibitors, (**II**) with NO_2_^−^-N (100 mg L^−1^) addition only, and (**c**) with NO_2_^−^-N (100 mg L^−1^) and inhibitors (ATU and NaClO_3_). HD, heterotrophic denitrification; AD, AOB denitrification.

Thus, three batch experiments were conducted: (I) no addition of nitrite or inhibitor, (II) the addition of nitrite, and (III) the addition of both nitrite and nitrification inhibitors (ATU and NaClO_3_). Three devices were assembled for these the batch experiments using an isotopic tracer; then, a 1-L mixture containing 100 mg wet weight of biofilm and 900 ml of wastewater (NH_4_^+^-N: 9.7 mg L^−1^; NO_2_^−^-N: 1.8 mg L^−1^; and NO_3_^−^-N: 23.6 mg L^−1^) from the SBBR were introduced into a sealed Erlenmeyer flask, and then, NaNO_2_, ATU, and NaClO_3_ were added to the effluent at concentrations of 100.0 mgN L^−1^, 10.0 mg L^−1^, and 1.0 g L^−1^, respectively. Helium gas was introduced into the wastewater to ensure anaerobic conditions. The solution and off-gas in the devices were sampled every 6 h for 24 h, and the concentrations of NH_4_^+^-N, NO_2_^−^-N, NO_3_^−^-N and TN in the wastewater were measured to investigate the characteristics of N transformation. The N_2_O emissions were also detected to identify the contributions of AOB denitrification and HD. The amount of N_2_O emissions can be described as follows: II–I, the sum of AOB denitrification and HD; III–I, HD; and (II–I)–(III–I), AOB denitrification (Fig. 5).

### Physicochemical analysis

The concentrations of TN, NH_4_^+^-N, NO_2_^−^-N, and NO_3_^−^-N were measured using a flow injection analyser (HachQuickchem 8500S2, Hach Inc., Loveland, CO, USA). Alkalinityand biomass dry weight (mixed liquid suspended solids, MLSS) were measured according to standard methods for water and wastewater^43^. The concentration of N_2_O was determined with an Agilent 7820A gas chromatograph (Agilent Technology Inc., Santa Clara, CA, USA) according to Jia *et al*.^40^. The dissolved N_2_O in wastewater was determined using the head space gas method described by Tsuneda *et al*.^44^. The values of *R*_30/29_ for N_2_ and *R*_46/45_ for N_2_O were measured by isotope-ratio mass spectrometry (IRMS;MAT253, Thermo Finnigan LLC, San Jose, CA, USA) according to the method described by Cao *et al*.^45^. The N_2_O-N emission factors per TN converted during the interval i–i + 1 (h) and the whole process were calculated using Eqs 7 and 8, respectively:


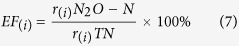



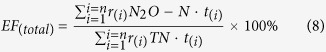


where r_(i)_N_2_O-N and r_(i)_TN represent the average rates of N_2_O emissions and TN removal, respectively during the interval i–i + 1 (h); and t_(i)_ is the duration of interval i–i + 1 (h).

### Microbial composition

To analyse the microbial composition in the one-stage autotrophic N removal process, biofilm from the SBBR was collected and centrifuged for to extract the DNA. The total genomic DNA was extracted using an E.Z.N.A.^®^ Soil DNA Kit (OMEGA Bio-Tek, Inc., Norcross, GA, USA), and the bacterial 16S rDNA genes of the biofilm were sequenced using Illumina MiSeq technology at the Shanghai Majorbio Bio-pharm Technology Co., Ltd. (Shanghai, China). Ultra-fast sequence analysis (USEARCH) was used to cluster the operational taxonomic units (OTUs) of a 16S DNA gene based on 97% similarity, and the statistical abundances of different OTUs in the samples reflect those of different microbial species. Then, the microbial composition was analysed according to sequencing information and data from the National Center of Biotechnology Information (NCBI) reference genome. Finally, microorganisms were classified as Anammox bacteria, AOB and HD bacteria based on the pathway of N metabolism. Simultaneously, the relative proportions of these microorganisms were calculated based on the OTU abundances.

## Additional Information

**How to cite this article**: Li, K. *et al*. Pathways of N removal and N_2_O emission from a one-stage autotrophic N removal process under anaerobic conditions. *Sci. Rep.* **7**, 42072; doi: 10.1038/srep42072 (2017).

**Publisher's note:** Springer Nature remains neutral with regard to jurisdictional claims in published maps and institutional affiliations.

## Figures and Tables

**Table 1:** Strategies for substrate addition in isotope batch experiments and the N removal performance.

Groups	Influent NH_4_^+^-N mg L^−1^	Influent NO_2_^−^-N mg L^−1^	Effluent TN mg L^−1^	Rate of TN removal mg (L h)^−1^
A	None	None	1.84	−0.08
B	100.0	None	98.31	0.07
C	100.0	100.0	60.42	5.82
